# Differences in HIV, STI and Other Risk Factors Among Younger and Older Male Sex Workers Who Have Sex With Men in Nairobi, Kenya

**DOI:** 10.3389/frph.2022.888403

**Published:** 2022-07-19

**Authors:** Nicholas Muraguri, Jerry O. Okal, Marleen Temmerman, Dunstan Mukoko, Helgar K. Musyoki, Peter Gichangi

**Affiliations:** ^1^Ministry of Health, Nairobi, Kenya; ^2^Population Council, Nairobi, Kenya; ^3^Director Women's Health, Faculty of Heath Sciences, Aga Khan University Hospital, Nairobi, Kenya; ^4^Independent Consultant, Nairobi, Kenya; ^5^Deputy Vice Chancellor Academic Affairs, Research and Extension, Technical University of Mombasa, Mombasa, Kenya

**Keywords:** male sex workers, men who have sex with men, HIV prevalence, STI prevalence, behavioral risk factors, Nairobi, Kenya

## Abstract

**Introduction:**

Previous surveys of male sex workers (MSW) in sub-Saharan Africa have not fully documented the HIV and sexually transmitted infections (STIs) rates and vulnerabilities by age category.

**Methods:**

The bio-behavioral survey of MSW in Nairobi, Kenya, utilized respondent-driven sampling to recruit MSW. Structured interviews captured MSW's behavioral aspects, and biological tests for HIV and other STIs.

**Results:**

Analysis of the two age categories, 18–24 years (younger MSW) and 25 years and above (older MSW), shows that of all participants, a significantly higher proportion of younger MSW (59.6% crude, 69.6% RDS-adjusted) were recruited compared to older MSW (40.4% crude, 30.4% RDS-adjusted, *P* < 0.001). Young male sex workers were more likely to report multiple sexual partnerships in the last 12 months and had multiple receptive anal intercourses (RAI) acts in the last 30 days than older MSW: 0–2 RAI acts (20.6 vs. 8.6%, *P* = 0.0300), 3–5 RAI acts (26.3 vs. 11.5, *P* < 0.001), and >5 RAI acts (26.3 vs. 11.5%, *P* < 0.01). Furthermore, younger MSW were significantly more likely to have 3–5 insertive anal intercourse (IAI) with a regular male sex partner in the last 30 days than older MSW (24.3 vs. 8.0%, *P* < 0.01). Younger MSW were also more likely to report other STIs [28.5% (95% CI: 19.1–40.4%)] than older MSW [19.0% (95% CI: 7.7–29.2%)]. However, older MSWs were more likely to be infected with HIV than younger MSW (32.3 vs. 9.9 %, *P* < 0.01).

**Conclusions:**

Owing to the high risk sexual behaviors, HIV and STIs risks among younger and older MSW, intensified and targeted efforts are needed on risk reduction campaigns and expanded access to services.

## Introduction

In most sub-Saharan African countries, HIV transmission mainly has been documented to mostly occur through heterosexual sex, but over the years, increasing evidence is showing that HIV infection also occurs through male-male anal sex (1). Worldwide, men who have sex with men (MSM) are at an increased risk of being infected with HIV than the general population because of the risks associated with unprotected anal sex, making it easy to acquire or transmit HIV (2). In Kenya, sexual intercourse between men is highly stigmatized, thereby making such sexual acts secretive, preventing open discussion that hinder, provision of targeted HIV and sexually transmitted infections (STI) services (1, 3). For the most part, the community, including religious groups, the political class, government officers and some health providers, still view MSM as abnormal and therefore oblivious of their health needs (1). Lack of recognition of MSM is compounded by limited information, inadequate data, and advocacy in Kenya and the sub-Saharan Africa region where on male-male sexual practices and related topics around their sexual behaviors, risk of HIV and STIs is rarely discussed.

The 2018 Joint United Nations Program on HIV/AIDS UNAIDS report in sub-Saharan Africa found that whereas the prevalence rate of HIV in the general population was between 0.1 and 19%, the rate of infection among MSM was often 13 to 17 times higher (4). Previous studies in Kenya have suggested high HIV and STI infection rates, including unprotected anal sex and increased numbers of sexual partners among male sex workers (MSW) and MSM (5). Exceptionally high rates of HIV infection were reported in the only known probability studies of MSW and MSM previously conducted in Nairobi and Mombasa (5–7).

There is evidence that more young men who have sex with other men (18–30 years) represent a growing proportion of new HIV and STI diagnoses (8). However, there have been no focused study and age-disaggregated data of male sex workers and the associated HIV and STI risks. More so, in Kenya and the sub-saharan Africa region, there is a dearth of probability-based estimates of HIV prevalence and associated risk factors among male sex workers.

In 2012, the Kenya National HIV/AIDS and STI Control Programme (NASCOP) and UNFPA conducted a surveillance survey of MSW who sell sex to other men in Nairobi. The results reported here are from a cross-sectional survey to estimate the prevalence of HIV, STIs, and document sexual behaviors among MSW. This study, to our knowledge, was the first surveillance survey that studied MSW and compared HIV and other risk factors among younger (18–24 years) and older (25 years and above) MSW.

## Methods

### Study Design and Setting

A cross-sectional survey was conducted from November 2012 to March 2013 at the NASCOP Voluntary Counseling and Testing (VCT) Center in Nairobi. Participants were recruited using respondent-driven sampling (RDS), a probability-based peer recruitment sampling method. RDS utilizes variant of chain referral sampling that employs a dual system of compensation. RDS draws on both Markov-chain theory and the theory of biased networks. To control the speed of sampling and to reach the target sample size, during survey implementation we closely observed waves of recruitment, the number of coupons issued per recruiter, as well as coupon activation and expiration dates and compensation. Recruitment was closely monitored to attain equilibrium in key variables to ensure that sample contains sufficient diversity with respect to characteristics that are known to exist in the target population. A detailed description of RDS methods is beyond this protocol's scope and can be found elsewhere (9–11). Recruitment started with 6 “seeds” who were taken through the study procedures and asked to recruit their male sex workers peers from their social networks (3 peers each). Seeds were identified through formative research (focus group discussions and key informants with groups of men selling sex to other men) and diversified by age, engagement in sex work, and marital status. Seeds and consequent recruits were compensated for up to 3 recruits. Recruitment continued until the required sample size was achieved.

### Study Participants

Study eligibility was considered for male participants who were 18 years or older, residents of Nairobi or the metropolis area and who had sold sex (anal or oral) with another man within 6 months preceding the interviews, and those who consented to participate in the study. The recruitment of study participants was tracked via an RDS coupon manager software. A biometric tool that has electronic fingerprint recognition software was used to detect duplicate recruits, confirm participant's proper ownership of coupons, and help identify recruits during follow-up. The study protocol was reviewed and approved by KNH-UoN Ethics and Research Committee.

### Data Collection

One-on-one structured interviews were administered using handheld computers and took approximately 35–50 min to complete. The survey questions covered demographic characteristics, alcohol and drug use, sexual risk behaviors, HIV testing, STIs, and experience with violence and discrimination. Data were collected from November 2012 to March 2013 at the NASCOP Voluntary Counseling and Testing (VCT) Center in Nairobi.

### Laboratory Methods

HIV testing was conducted with Determine and Unigold rapid tests (Determine; Abbott Laboratories, Abbott Park, IL; Unigold; Trinity Biotech plc, Bray, Ireland) and Bioline (Standard Diagnostics Inc., Gyeonggi-Do, South Korea) as a tiebreaker for discordant results. Rectal and urine samples were collected from all participants and tested for genital and rectal chlamydia and gonorrhea using Roche Amplicor test kits (Roche Molecular Diagnostics, Pleasanton, CA). Rectal swabs that tested positive for gonorrhea were confirmed using the Aptima2 ComboAssay (Gen-Probe, San Diego, CA). For syphilis, venous blood was collected and tested using the rapid plasma reagent assay, and positives were confirmed with the Treponema palladium hemagglutination assay test (Human Diagnostics Worldwide, Wiesbaden, Germany). STI tests and HIV polymerase chain reaction were conducted for quality assurance at the University of Nairobi Institute of Tropical and Infectious Diseases Laboratory. Gen-Probe confirmatory gonorrhea testing of positive results was performed at the University of Nairobi Institute of Tropical and Infectious Diseases Laboratory.

### Statistical Analysis

We compared sociodemographic and behavioral characteristics, HIV testing, and HIV and STI prevalence and exposure to violence and discrimination among young MSW aged 18–24 and older MSW aged 25 and above who 1) reported selling sex in the past 6 months and/or 2) reported sex work as their “main” occupation. IBM SPSS Statistics (version 19) and Stata (version 10.1; StataCorp, College Station, TX) were used for the management and analysis of data. RDS Analysis Tool (RDSAT Version 7.1.46) was used to produce population-level proportions and 95% confidence intervals weighted for network sizes and recruitment patterns. To test statistical differences between young male sex workers and older sex workers, we used the adjusted point estimates and their confidence intervals to calculate a z-score and its associated *P*-value; the *z*-test has been applied to RDS data previously.

## Results

There were six (7) seed recruiters in the study. The analysis of the socio-demographic characteristics of the seeds was done separately and excluded from entire analysis of survey respondents per the RDS requirements. The socio-demographic and sexual behavior analysis of the 6 seeds recruiters shows that 4 seeds were in the 18–24 years age band (66.9%), and 2 were 25 years or older (33.1%). Most seeds (*n* = 5, 83.3%) attained none/complete primary = 5 (83.3%) and above secondary level (*n* = 16.7%). All six seeds (100%) were single/never married and had sexual debut with a man at 15 years or older. Among all the seeds, the number of regular male partners among all sexual partners in the past 12 months was 0–2 = 2 (33.3%); 3–5 = 2 (33.3%), >5 = 2(33.3%); the number of reported RAI acts with regular male sex partners in the last 30 days was 0–2 = 3 (50%); 3–5 = 1(16.7%); >5 = 2(33.3%); the number of reported IAI acts with regular male sex partners in the last 30 days was 0–2 = 3(50%); 3–5 = 1(16.7%). None of the 6 seeds recruiters reported URAI or UIAI with last male sexual partner. And, only one out of the six seeds reported >5 insertive vaginal sex acts with regular female partners in the last 30 days.

Among the survey respondents, a total of 282 MSW who reported selling sex were recruited through 6 seeds to participate in the study. A significantly higher proportion of younger MSW–aged 24 years and below–(59.6% crude, 69.6% RDS-adjusted) were recruited compared to older MSW–aged 25 years and above (40.4% crude, 30.4% RDS-adjusted, *P* < 0.001). The youngest MSW was aged 18 years and the oldest 68 years.

A significant proportion of male sex workers who were 18–24 years were more likely to be single/never married than those above 25 years or older MSW (63.4 vs. 17.3%, *P* < 0.001) and to have completed secondary school education (37.2 vs. 12.9%, *P* < 0.001). In comparison, a significant proportion of young MSW had greater odds of reporting none/completed primary education (24.7 vs. 14.7%, *P* = 0.033) and completed secondary education than MSW aged above 25 years (37.2 vs. 12.9%, *P* < 0.001). Less than ten percent of younger MSW (7.3%) and older MSW (3.1%) had a postsecondary level of education (Table 1).

**Table 1:** Socio-demographic characteristics of MSW who sell sex to other men in Nairobi, Kenya.

**Characteristic**	**MSW ages 18-24 (*****n** **=*** **162)**		**MSW ages 25 and above (*****n** **=*** **120)**		
	**n (Crude %)**	**Adjusted % (95% CI)**	**n (Crude %)**	**Adjusted % (95% CI)**	***Z (p-value)***
**Age group** (years)	140 (59.6)	69.6 (61.9–79.1)	95 (40.4)	30.4 (20.9–38.1)	**6.317 (<0.001)**
**Marital status (with women)**					
Single, never married	143 (50.7)	63.4 (55.0–71.9)	80 (28.4)	17.3 (11.1–22.2)	**5.673 (<0.001)**
Previously married	12 (4.3)	4.5 (2.0–9.3)	22 (7.8)	9.9 (4.7–15.2)	1.1163 (0.264)
Currently married	7 (2.5)	1.9 (0.1–4.6)	18 (6.4)	3.0 (1.5–5.6)	0.2990 (0.765)
**Education**					
None/Completed primary	57 (20.2)	24.7 (17.6–32.1)	57 (20.2)	14.7 (8.9–20.2)	**2.1323 (0.033)**
Completed Secondary	82 (29.1)	37.2 (30.1–47.9)	46 (16.3)	12.9 (8.0–18.3)	**4.6318 (<0.001)**
More than secondary	23 (8.2)	7.3 (2.7–12.4)	17 (6.0)	3.1 (1.0–4.5)	1.5965 (0.1104)

*The bold values (statistic and P-value) indicate statistically significant differences at P < 0.05*.

Most younger MSW were more likely to report being single/never married compared to MSW aged 25 years and above (63.4 vs. 17.3 %, *P* < 0.001). However, while most MSW aged 25 and older reported reported being previously (4.5 vs. 9.9%, *P* = 0.264) or currently married (1.9 vs. 3.0, *P* = 0.765), the differences were non-significant.

More than a third, 77.5%, of all the MSW, reported drinking alcohol (182/235). Among those who took alcohol, a significant proportion of the 18–24-year-olds (58.0%) scored 0–19 on the AUDIT score depicting low to possible hazardous use of alcohol compared to men aged 25 years and above (20.4%, *P* < 0.001). An almost similar proportion of both young and old MSW scored 20–40 on the AUDIT score, implying possible alcohol dependence (11.4 vs. 10.2%, *P* = 0.7507). About half of all the MSW reported taking illicit drugs in the past 12 months, with a significantly higher proportion of younger MSW reporting the use of illegal drugs than the older MSW (42.3 vs. 15.0%, *P* < 0.001) (Table 2).

**Table 2:** Alcohol and drugs characteristics.

**Characteristic**	**MSW ages 18–24 (*****n** **=*** **162)**		**MSW ages 25 and above (*****n** **=*** **120)**		
	**n (Crude %)**	**Adjusted % (95% CI)**	**n (Crude %)**	**Adjusted % (95% CI)**	***Z (p-value)***
***Alcohol dependence (AUDIT score)***					
0 to 19 - low to possible hazardous use	135 (47.9)	58.0 (50.0–67.3)	76 (27.0)	20.4 (13.1–26.0)	**6.8299 (<0.001)**
20 to 40 - possible alcohol dependence	27 (9.6)	11.4 (6.3–17.9)	44 (15.6)	10.2 (5.4–14.6)	0.3177 (0.7507)
**Any illicit drug tried in past 12 months**	103 (36.5)	42.3 (34.0–52.7)	66 (23.4)	15.0 (8.1–19.6)	**4.8747 (<0.001)**

*The bold values (statistic and P-value) indicate statistically significant differences at P < 0.05*.

A substantial proportion of young MSW had their sexual debut with another man when they were 15 years or below (10.2 vs. 1.2%, *P* < 0.01). Similarly, those who had their first sexual debut when they were 15 years or older were younger MSW (60.6 vs. 28.0%, *P* < 0.001).

Young male sex workers were significantly more likely to have multiple male sexual partners in the 12 months preceding the interview. Similarly, more young MSWs than the older ones reported having multiple sexual partners in the last 12 months: 0–2 (19.0 vs. 8.5%, *P* = 0.0422), 3–5 (33.7 vs. 9.8%, *P* < 0.001) and >5 (22.7 vs. 6.2%, *P* < 0.001).

Young male sex workers were significantly more likely to have had multiple receptive anal intercourse (RAI) acts in the last 30 days than older MSW. Younger MSW compared to older MSW reported the following frequencies of RAI acts in the last 30 days: 0–2 (20.6 vs. 8.6%, *P* = 0.0300), 3–5 (26.3 vs. 11.5, *P* < 0.001), and >5 (26.3 vs. 11.5%, *P* < 0.01). A non-significant but high proportion of young MSW reported unprotected RAI with the last male partners than the older MSW (23.5 vs. 11.2%, *P* = 0.0619). Most young MSW's were significantly more likely to have 3–5 insertive anal intercourse (IAI) with a regular male sex partner in the last 30 days than older MSW (24.3 vs. 8.0%, *P* < 0.01). A non-significant but high proportion of older MSW reported unprotected IAI with the last male partners than the younger MSW (23.4 vs. 8.7%, *P* = 0.3325). Further, young male sex workers were significantly more likely to have had multiple insertive vaginal sex acts with regular female sexual partners in the last 30 days. The reported frequency of insertive vaginal sex acts among younger and older MSW were as follows: 0–2 (34.7 vs. 0.6%, *P* = 0.0176) and 3–5 (31.4 vs. 10.8%, *P* = 0.0487) (Table 3).

**Table 3:** Sexual behavior.

**Characteristic**	**MSW ages 18-24 (*****n** **=*** **162)**		**MSW ages 25 and above (*****n** **=*** **120)**		
	**n (Crude %)**	**Adjusted % (95% CI)**	**n (Crude %)**	**Adjusted % (95% CI)**	Z (p-value)*
**Age at sexual debut with a man**					
Less than 15 years old	18 (8.1)	10.2 (5.30–16.6)	7 (3.20)	1.2 (0.10–3.10)	**3.018 (< ** **0.01)**
15 years or older	114 (51.4)	60.6 (52.9–70.7)	83 (37.4)	28.0 (18.2–34.4)	**5.310 (<0.001)**
**Number of regular male partners among all sexual partners in last 12 months**					
0–2	34 (18.3)	19.0 (11.3–28.8)	21 (11.3)	8.5 (3.8–14)	**2.0322 (0.0422)**
3–5	49 (26.3)	33.7 (25.0–44.5)	29 (15.6)	9.8 (5.1–15.3)	**4.2572 (<0.001)**
>5	35 (18.8)	22.7 (16.2–32.8)	18 (9.7)	6.2 (2.6–10.1)	**3.5507 (<0.001)**
**Number of reported male RAI acts in last 30 days**					
0 to 2 acts	26 (17.1)	20.6 (13.2–33.1)	17 (11.2)	8.6 (2.5–11.1)	**2.1698 (0.0300)**
3 to 5 acts	35 (23.0)	26.3 (15.8–34.2)	17 (11.2)	6.7 (3.7–14.0)	**3.5550 (<0.001)**
Over 5 acts	33 (21.7)	26.3 (18.2–37.2)	24 (15.8)	11.5 (5.7–17.2	**2.6122 (<0.01)**
**Reported any URAI with last male partners**	16 (20.3)	23.5 (15.1–37.3)	12 (15.2)	11.2 (2.3–15.5)	1.8668 (0.0619)
**Number of reported IAI acts with regular male sex partners in last 30 days**					
0 to 2 acts	19 (19.6)	26.5 (15.4–44.1)	15 (15.5)	15.6 (8.7–25.9)	1.2945 (0.1955)
3 to 5acts	19 (19.6)	24.3 (9.7–30.8)	12 (12.4)	8.0 (2.0–12.9)	**2.6904 (<0.01)**
Over 5 acts	14 (14.4)	15.9 (8.0–29.3)	18 (18.6)	9.7 (3.7–16.5)	0.9780 (0.3280)
**Reported any UIAI with last male partners**	0.0 (0.0)	8.7 (0.0–32.8)	5 (33.3)	23.4 (4.0–53.6)	0.9690 (0.3325)
**Number of insertive vaginal sex acts with regular female sexual partners in last 30 days**					
0–2 acts	6 (20.1)	34.7 (7.4–63.3)	1 (3.3)	0.6 (0.0–1.7)	**2.3731 (0.0176)**
3–5 acts	8 (26.7)	31.4 (14.3–50.8)	5 (16.7)	10.8 (0.1–18.7)	**1.9712 (0.0487)**
Over 5 acts	5 (16.7)	11.5 (0.0–28.2)	5 (16.7)	11.1 (0.0–27.3)	0.0399 (0.9681)

*The bold values (statistic and P-value) indicate statistically significant differences at P < 0.05*.

More than half of the young MSW had ever been counseled or tested for HIV than the older MSW (59.7 vs. 24.6%, *P* < 0.001). A similar proportion of young MSW compared to older MSW had been tested for HIV within the past 12 months (59.2 vs. 24.5%, *P* < 0.001).

Overall, HIV prevalence among MSW in this study (both young sex workers and old sex-workers alike) was 16.4% (95% confidence interval: 9.0 to 24.2). A significantly higher proportion of older male sex workers were HIV infected [32.3% (95% CI: 15.1–43.5)] than young male sex workers [9.9% (95% CI: 3.7–16.5)], *P* < 0.01. Prevalence of syphilis, gonorrhea (rectal), or chlamydia did not significantly differ between the two age bands. However, the prevalence of testing positive for one or more of these 3 STIs combined among young MSWs [28.5% (95% CI: 19.1–40.4)] was higher than among older MSW [19.0% (95% CI: 7.7–29.2), although the difference was not significant; *P* = 0.2207)].

Partition analysis by type of anal sex (both receptive or insertive sex) and STI showed that rectal chlamydia infections were significantly higher among participants practicing RAI [11.5%, (95% CI: 3.5–23.9)] than those practicing insertive anal sex intercourse [9.4% (95% CI: 0.0–29.1), *P* < 0.001]. There was no significant difference in rectal gonorrhea infection among young and older MSW (4.7 and 6.0% respectively), *P* = 0.8704 (Table 4).

**Table 4:** HIV, STI, and Hep B&C testing and prevalence.

**Characteristic**	**MSW ages 18-24 (*****n** **=*** **162)**		**MSW ages 25 and above (*****n** **=*** **120)**		
	**n (Crude %)**	**Adjusted % (95% CI)**	**n (Crude %)**	**Adjusted % (95% CI)**	Z (p-value)*
**HIV counseling and testing**					
Ever counseled or tested for HIV	121 (51.5)	59.7 (52.7–69.3)	83 (35.3)	24.6 (17.1- 32.2)	**6.13134 (<0.001)**
Tested for HIV in past 12 months	118 (51.3)	59.2 (52.6–69.9)	81 (35.2)	24.5 (16.6–32.6)	**5.7723 (<0.001)**
**HIV, STI and Hep B prevalence and**					
HIV	13 (7.4)	9.9 (3.7–16.5)	25 (14.3)	32.3 (15.1–43.5)	**2.81871 (<0.01)**
Syphilis	1 (0.6)	0.3 (0.0–1.0)	0 (0.0)	0.0 (0.0–0.0)	1.17598 (0.2396)
Gonorrhea (any)					
Gonorrhea (Rectal)	11 (6.4)	11.4 (3.9–18.5)	3 (1.7)	5.0 (5.0–5.0)	1.71833 (0.0857)
Chlamydia (any)					
Chlamydia (Rectal)	11 (6.4)	10.8 (4.9–20.6)	6 (3.5)	7.4 (1.6–15.0)	0.64569 (0.5185)
Chlamydia (Genital)	6 (3.5)	8.2 (2.3–14.4)	5 (2.9)	9.3 (0.7–19.6)	0.19214 (0.8476)
Any STI (excluding HIV)	28 (26.4)	28.5 (19.1–40.4)	13 (19.7)	19.0 (7.7–29.2)	1.22475 (0.2207)
Hep B	1 (0.9)	1.1 (0.0–6.6)	3 (1.8)	2.9 (0.0–5.9)	0.79703 (0.42543)

*The bold values (statistic and P-value) indicate statistically significant differences at P < 0.05*.

Although there were observed low rates of victimization to verbal abuse, a higher proportion of young MSW reported being targeted with verbal abuse (14.9 vs. 9.9%, *P* = 0.1675), physical (13.2 vs. 10.1%, *P* = 0.3674), and sexual violence (4.0 vs. 3.6%, *P* = 0.8418) than older MSW (Table 5).

**Table 5:** Violence and discrimination.

**Characteristic**	**MSW ages 18-24 (*****n** **=*** **162)**		**MSW ages 25 and above (*****n** **=*** **120)**		
	**n (Crude %)**	**Adjusted % (95% CI)**	**n (Crude %)**	**Adjusted % (95% CI)**	Z (p-value)*
**Violence and discrimination in the past year**					
Verbally assaulted	33 (14.6)	14.9 (9.5–21.3)	34 (15.0)	9.9 (3.9–11.8)	1.3802 (0.1675)
Physically assaulted	31 (13.2)	13.2 (8.5–19.2)	34 (14.5)	10.1 (4.4–12.6)	0.90142 (0.3674)
Sexually assaulted	10 (4.4)	4.0 (1.3–8.0)	10 (4.4)	3.6 (0.8–4.9)	0.19962 (0.8418)
Refused services	10 (4.3)	3.5 (1.1–6.4)	11 (4.7)	2.6 (**)	

** = Statistic could not be computed.

## Discussion

This survey was the second probability-based surveillance survey of men who have sex with men (MSM) in Kenya. The initial probability survey targeted MSM and determined the existence of MSW by asking a set of questions on selling sex for money or goods to another man (5). However, in this study, recruitment into the study was based entirely on participants' engagement in male-male sex work. Among all MSW in this study, HIV prevalence was 16.4%, while HIV prevalence among older male sex workers (32.3%) was more than thrice that of young sex workers (9.9%). The rates of HIV infection among younger MSW (9.9%) are much lower compared to those documented among MSW in the 2010 study (5). However, the rates of HIV infection among older MSW (32.3%) are higher than the rates of HIV infection observed in the initial first probability survey that recruited both MSW and MSM (26.3%) (5). More so, as expected, the rates of HIV infection among non-sex workers were much lower in 2010, at 12.2%. Although there was some variance in the target sample for the 2010 and 2012 studies, the observed differences in HIV infection rates demonstrate the importance of disaggregating the data by age and sex work status. This illustrates the importance of being aware of active/inactive sub populations in research especially among key and priority populations like men who have sex to men. At the same time, the variable HIV infection rates point toward two scenarios: the possibility of a maturing epidemic owing to the high infection rates among older MSW and likely lower HIV incidence rates (owing to younger sex workers reported lower risk behaviors viz-viz their HIV prevalence).

In this study, younger MSW reported multiple sexual partnerships—including unprotected receptive anal intercourse, unprotected insertive vaginal sex acts, and possible hazardous alcohol use compared to older MSW. On the other hand, a significant proportion of older MSW had unprotected insertive anal intercourse. Given the risks of receptive anal intercourse the likely scenario would be increased risk of HIV which is not observed among younger MSW. Potentially, lower rates of HIV point toward the existing HIV prevention efforts, especially those targeting key and priority populations like MSW and men who have sex with men in general.

Prevalence of syphilis, gonorrhea, or chlamydia did not significantly differ between the age groups. However, the risk of acquiring rectal chlamydia was higher among participants practicing RAI. However, despite these varying risk behaviors (among younger and older MSW), the HIV infection rates among the older MSW were significantly higher. As noted earlier, a maturing HIV epidemic (people living with HIV longer) and increased access to HIV prevention efforts especially ARV treatment and PrEP by older MSW is likely changing the course of the epidemic. The net effect of therapeutics and HIV prevention treatments means that more people are living longer and have healthier lives.

Most MSWs, especially the younger ones, reported taking alcohol, possibly as part of socializing with other people including MSW. Yet, being intoxicated with alcohol and drugs has been found to increase the likelihood of having unprotected sex, having a higher number of sexual partners, and increases the risk of HIV transmission. For instance, in India, a study among men who have sex with men found a link between alcohol, increased sexual risk behavior, and HIV acquisition (12–14). In comparison to the findings from India, our study demonstrated increased risk of HIV among younger MSW but lower rates of HIV which requires further investigation.

Although younger MSW have substantial lower rates of HIV infection, they engage in high risk behaviors place them at heightened risk of HIV and STIs. Hence, intensified efforts are needed on risk reduction campaigns and expanded access to services to mitigate the risks. While among older MSW, targeted testing should be made a priority as most male-male sexual practices are largely hidden and may account for the highest percentage of MSW with undiagnosed HIV infection. Increasing HIV prevention and testing services for older MSM can help diagnose HIV infection sooner, enable HIV positive MSW to be identified early, linked and enrolled in HIV treatment, and reduce the risk of HIV transmission to others (15). To avert the most significant number of infections and improve health outcomes, MSW should be tested or self test whenever they perceive themselves to be at risk of HIV infection or when they are exposed to high-risk situations. Those refusing testing or those found to be negative should be given further counseling and provided with HIV prevention services, such as PrEP and other prevention options (16, 17). Not testing for HIV means that many men who have sex with men might end up being unaware of their HIV status and may be uninformed of the need to take protective measures to prevent onward transmission to others.

The study findings are subject to at least four limitations. There are inherent limitations to RDS sampling methodology, and providing an accurate representative estimate might prove to be a challenge (18). The findings from this study in Nairobi reached a sub-sample of MSW and are possibly not generalizable to MSW living in other parts of Kenya. Also, the study relied on self-report for all behaviors and demographics, and these data may be subject to social desirability bias. Finally, sex work, especially male sex work, is illegal and highly stigmatized in Kenya. Thus other potential participants might have declined participation in the study for being known to be selling sex and practicing sex with other men.

## Conclusions

Overall, although Kenya has made significant strides in HIV programming for MSM, more still needs to be done to address the structural barriers and biological risks. For example, there is a need to address the structural barriers (i.e have an enabling environment for services access, address stigma and discrimination especially within health care, and entrench advocacy and supportive laws and policies to promote the health of MSM) and. reach of biological interventions such as PrEP. Future HIV prevention programs and research for MSW must address age-related risks and vulnerabilities, structural barriers, and especially interventions that may decrease homophobia, violence, and stigma. These findings highlight the need to target both young and old MSW with appropriate interventions focused on HIV prevention, care, and treatment, including riskreduction counseling and screening and provision of PrEP to MSW at high risk for HIV acquisition. Promotion of safer sex practices and care and treatment will help improve positive health outcomes and reduce onward transmission to others, particularly if prevention efforts are tailored to specific age groups.

## Data Availability Statement

The raw data supporting the conclusions of this article will be made available by the authors, without undue reservation.

## Ethics Statement

The study protocol involving human participants was reviewed and approved by University of Nairobi/Kenya National Hospital Ethics Review Committee. The study participants provided their written informed consent to participate in this study.

## Author Contributions

NM, JO, MT, HM, and PG conceptualized the paper. NM and JO wrote the first draft of the paper. MT, DM, HM, and PG contributed in writing and reviewing different sections of the paper. JO and DM generated the data and conducted the analysis. NM and HM supported in data collection. All authors contributed to the article and approved the submitted version.

## Funding

This study was supported by Ministry of Health, Kenya.

## Conflict of Interest

The authors declare that the research was conducted in the absence of any commercial or financial relationships that could be construed as a potential conflict of interest.

## Publisher's Note

All claims expressed in this article are solely those of the authors and do not necessarily represent those of their affiliated organizations, or those of the publisher, the editors and the reviewers. Any product that may be evaluated in this article, or claim that may be made by its manufacturer, is not guaranteed or endorsed by the publisher.
